# Impact of inorganic iron and haem on the human gut microbiota; An *in vitro* batch-culture approach

**DOI:** 10.3389/fmicb.2023.1074637

**Published:** 2023-02-23

**Authors:** Andrea Monteagudo-Mera, Arvindkumar Shalunkhe, Amro Duhduh, Gemma E. Walton, Glenn R. Gibson, Dora I. Pereira, Anisha Wijeyesekera, Simon C. Andrews

**Affiliations:** ^1^School of Biological Sciences, University of Reading, Reading, United Kingdom; ^2^Department of Food & Nutritional Sciences, University of Reading, Reading, United Kingdom; ^3^Faculty of Applied Medical Science, Jazan University, Jazan, Saudi Arabia; ^4^Vifor Pharma UK Limited, London, United Kingdom

**Keywords:** gut microbiota, inorganic iron, haem, batch cultures, *in vitro* models

## Abstract

Although iron is an essential nutrient for humans, as well as for almost all other organisms, it is poorly absorbed (~15%) from the diet such that most passes through the upper gut into the large intestine. The colonic microbiota is thus exposed to, and potentially influenced by, such residual iron which could have an impact on human health. The aim of the research described here is to determine how the major forms of dietary iron (inorganic iron and haem) influence metabolic activity and composition of the human gut microbiota by utilizing an *in vitro* parallel, pH-controlled anaerobic batch culture approach. Controlled iron provision was enabled by the design of a ‘modified’ low-iron gut-model medium whereby background iron content was reduced from 28 to 5 μM. Thus, the impact of both low and high levels of inorganic and haem iron (18–180 μM and 7.7–77 μM, respectively) could be explored. Gut-microbiota composition was determined using next generation sequencing (NGS) based community profiling (16S rRNA gene sequencing) and *flow*-fluorescent *in situ* hybridization (FISH). Metabolic-end products (organic acids) were quantified using gas chromatography (GC) and iron incorporation was estimated by inductively coupled plasma optical emission spectroscopy (ICP-OES). Results showed that differences in iron regime induced significant changes in microbiota composition when low (0.1% w/v) fecal inoculation levels were employed. An increase in haem levels from 7.7 to 77 μM (standard levels employed in gut culture studies) resulted in reduced microbial diversity, a significant increase in *Enterobacteriaceae* and lower short chain fatty acid (SCFA) production. These effects were countered when 18 μM inorganic iron was also included into the growth medium. The results therefore suggest that high-dietary haem may have a detrimental effect on health since the resulting changes in microbiota composition and SCFA production are indicators of an unhealthy gut. The results also demonstrate that employing a low inoculum together with a low-iron gut-model medium facilitated *in vitro* investigation of the relationship between iron and the gut microbiota.

## Introduction

Iron is an essential micronutrient for almost all living organisms. Its redox properties, range of spin states adopted and abundance largely explain iron’s importance in numerous biological processes where it serves as a co-factor for proteins (Crichton, 2016). Humans obtain iron exclusively from their diet but absorb it poorly (typically, ~15% of dietary iron) with variability being exhibited as a result of various dietary- and health-related factors (Hallberg and Hulthén, 2000). Such poor absorption is a major contributory factor to a high prevalence of iron-deficiency anaemia, estimated at 1.62 billion people globally (De Benoist et al., 2008). A key dietary factor influencing absorption is the form of iron present in the diet, with two major types, haem and inorganic (or non-haem) iron, recognized. Haem-iron (mainly found in meat products) has a relatively-high absorption rate of 20%–30% whereas inorganic iron (present in plant-based foods or iron-fortified products) has a much lower absorption rate, typically less than 10% (Hallberg et al., 1979; Collings et al., 2013). However, haem iron is less prevalent in the diet (10%–15% of total iron in an omnivorous diet) than inorganic iron (Bjorn Rasmussen et al., 1974) and as high-haem diets have been associated with enhanced risk of colorectal cancer and cardiovascular disease, haem may not be suitable as a dietary supplement (Bastide et al., 2011; Fang et al., 2015).

Iron absorption from the diet largely occurs in the duodenum and proximal jejunum in a fashion that is responsive to body-iron and -inflammation status (Nicolas et al., 2002; Schaible and Kaufmann, 2004; Frazer and Anderson, 2005). Since the majority of dietary iron is unabsorbed during transit through the small intestine, most remains available to the resident gut microbiota of the colon which carries the greatest bacterial density (Savage, 1977) and also, by inference, is the site where the overall demand of the gut microbiota for iron is greatest. Indeed, nearly all bacteria require iron for growth although members of the *Lactobacillus* genus, common and beneficial members of the gut microbiota, are notable exceptions due to the deployment of iron-alternatives such as manganese and cobalt (Imbert and Blondeau, 1998). In addition, *Bacteroides* species, another important component of the gut microbiota, are considered haem auxotrophs and thus have an absolute requirement for haem (or inorganic iron combined with protoporphyrin) (Rocha and Smith, 2010). Previous studies have indicated that iron availability to the gut microbiota is limited such that competition is an important colonization factor (Merrell et al., 2002; Naikare et al., 2006). Such iron-competition in the gut is supported by studies showing that inactivation of bacterial iron-acquisition systems results in failure to effectively colonize the gut (Stojiljkovic et al., 1993; Tsolis et al., 1996; Velayudhan et al., 2000). Iron availability can also have a dramatic influence on the expression of bacterial genes, including those involved in pathogenicity, stress resistance and energy metabolism (Kortman et al., 2012; Frawley et al., 2013). Therefore, the forms, bioavailability and overall levels of unabsorbed iron within the colon would be expected to influence composition and activity of the colonic microbial ecosystem. Indeed, such influences have been reported from both *in vitro* and *in vivo* studies, and common changes induced by inorganic-iron supplementation include the reduction of beneficial *Bifidobacterium* and *Lactobacillus* spp. levels, and promotion of potentially harmful *Enterobacteriaceae* spp. (Mevissen-Verhage et al., 1985; Zimmermann et al., 2010; Dostal et al., 2013, 2015; Jaeggi et al., 2015; Kortman et al., 2016; Parmanand et al., 2019). Bioavailability of iron in the gut can also be influenced by host defence factors lactoferrin and lipocalin-2, which act as a ferric-iron chelator and ferri-siderophore-sequestering agent, respectively, and are released in response to inflammation (Buderus et al., 2015).

Although several previous studies have investigated the impact of iron on the human gut microbiota, no consistent findings have emerged (Yilmaz and Li, 2018; Seyoum et al., 2021), partly due to differences in the approaches applied. Furthermore, studies on animal models such as rodents (Bastide et al., 2011; Dostal et al., 2012a; Constante et al., 2017) are unlikely to provide results that are closely reflective of humans (Zimmermann et al., 2010; Tang et al., 2017). The aim of the study described here is to address this issue by employing human gut-microbiota batch cultures since these can be operated in parallel under identical conditions with precise control of metal composition and offer a relatively rapid and well-established method for investigation of the impact of dietary factors on the gut microbiota (Frost et al., 2014; Liu et al., 2016; Harris et al., 2020). However, up until now, such systems have been little used for studies involving the precise control of micronutrients. In this work, initially, the culture growth medium and inoculation conditions were optimized to reduce background iron content to a minimal level and raise the potential for any impact of iron regime to develop. Following this, effects of haem and inorganic iron (at different concentrations and ratios) on the gut microbiota were explored revealing nonbeneficial haem-induced changes that were largely reversed by inclusion of inorganic iron. These results thus demonstrate utility of the adapted *in vitro* batch gut culture system described here as an investigative tool for dietary-iron/gut-microbiota interaction studies as well as negative effects of haem.

## Materials and methods

### Fecal inoculum preparation

Anaerobic batch-culture fermentation vessels were inoculated with non-pooled fecal samples provided by three donors (one male, two females; age 25–39 years). The donors were healthy, omnivore volunteers with no-history of anemia, free of known metabolic and gastrointestinal diseases, and had not received any antibiotic or probiotic treatment for at least 6 months prior to the experiment. Ethical approval for utilizing fecal samples from healthy volunteers was obtained from University of Reading University Local Research Ethics Committee (UREC 1520). Donors were healthy, with no metabolic or gastrointestinal conditions and had not received any antibiotic, probiotic, or prebiotic interventions for at least 6 months prior to the experiment. Fecal samples were collected in clean plastic containers that were placed in anaerobic jars containing AnaeroGen sachets (Oxoid, Basingstoke, UK) (<0.1% O_2_) and were used within 2 h of collection. Fecal samples were diluted in Phosphate Buffered Saline (PBS) at pH 7.0 at 1 or 10% (w/v) (depending on the inoculum concentration tested) in strainer bags, to enable removal of large particles (Seward, Worthing, UK), and homogenized using a Stomacher 400 (Seward, Worthing, UK) for 2 min at 200 rpm. Resulting fecal slurries were used to inoculate the batch culture systems at 10% of culture volume such that the final fecal concentrations in the vessels were 0.1 and 1% (v/v) for inoculations with 1 and 10% (w/v) fecal slurries, respectively.

### Gut model medium (GMM) and ‘modified’ gut model medium (mGMM)

The compositions of standard gut model medium (GMM) described previously by Macfarlane et al. (1998) and modified gut model medium (mGMM) used during this study are shown in Supplementary Table S1. Iron sources applied to the medium were FeSO_4_ and haem (ferriprotoporphyrin IX chloride dissolved in 1 M NaOH; Sigma). Total iron content of standard GMM was 123 μM (28 μM background iron content, plus 18 μM FeSO_4_ and 77 μM haem supplements). To achieve iron restriction, an iron-limited (≤5 μM) version of GMM was formulated, which was designated as ‘modified GMM’ (mGMM). To ensure low-iron status of mGMM, the iron content of every component of GMM was assessed by inductively coupled plasma-optical emission spectroscopy (ICP-OES). Thus, mGMM excluded the major background iron contributors of GMM: yeast extract, tryptone and mucin (contributing 1.33, 2.87 and 19.79 μM Fe, respectively; Table S1). Tryptone was replaced by peptone and yeast extract was replaced with a defined mineral and vitamin solution (Wolin et al., 1963) (Supplementary Table S1).

### *In vitro* batch cultures (200 ml) in GMM with/without haem and inorganic iron supplementation

**Figure 1:**
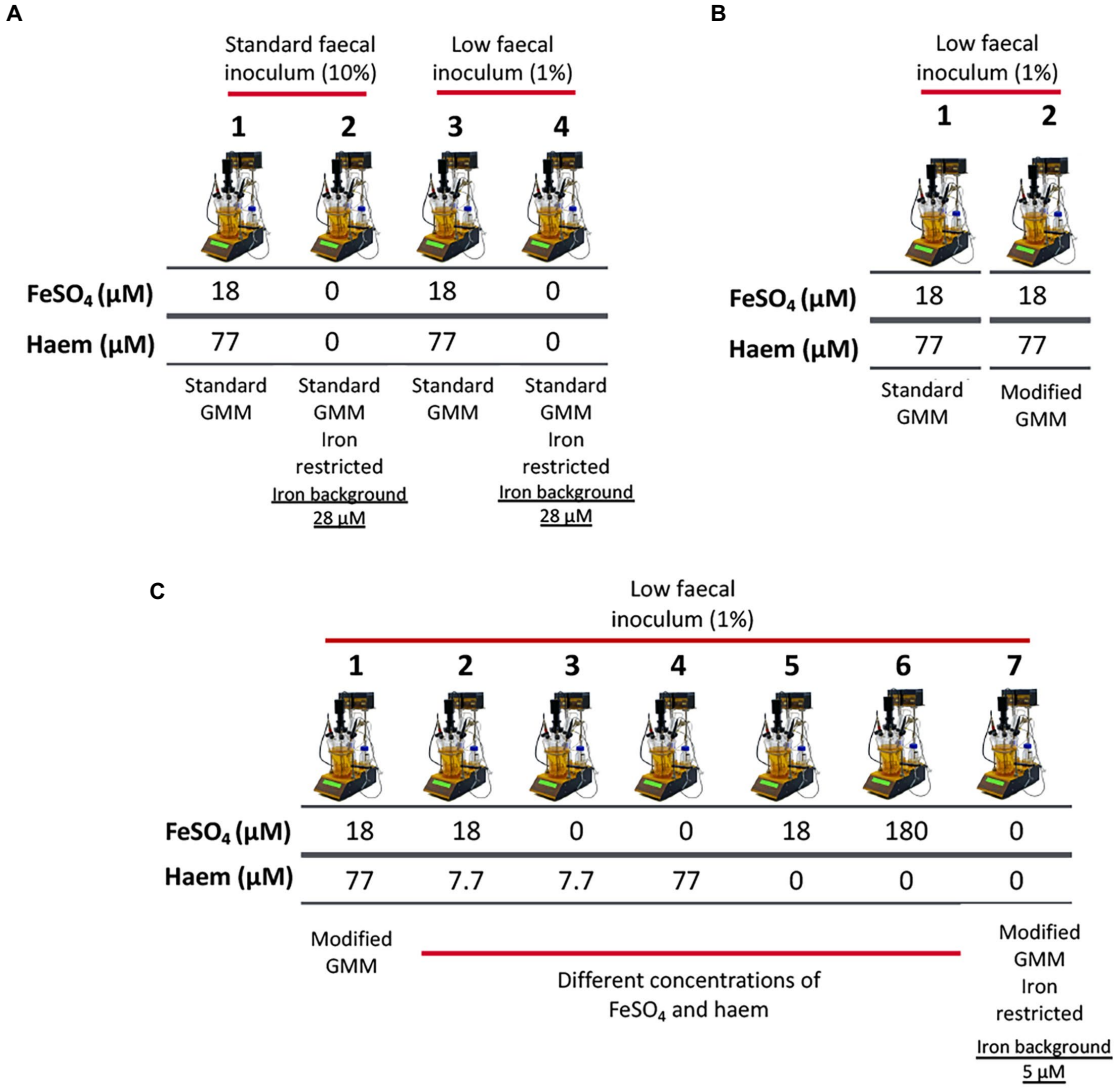
Schematic representation of experimental conditions employed in single-stage anaerobic batch gut-culture systems. **(A)** The impact of iron sources (FeSO_4_ and haem) in ‘standard’ Gut Model Medium (GMM; 28 μM background iron) was assessed over 48 h of fermentation. In addition, two concentrations of faecal inocula were tested: 10% (w/v) (‘high’ faecal inoculum) and 1% (w/v) (‘low’ faecal inoculum). Each fermentation was performed in triplicate with 3 different healthy donors (*n* = 3). Technical duplicates were also performed for each condition. **(B)** Validation of the modified GMM (mGMM; 5 μM background iron), as developed for this study. Standard GMM and mGMM were compared during a 36 h fermentation period using a low concentration (1% w/v) of faecal inoculum. The fermentations were each performed six times with six different healthy donors (*n* = 6). **(C)** Different forms (haem and FeSO_4_) and concentrations of iron were used to supplement the low-iron (~5 μM Fe) mGMM, and low faecal inocula (1% w/v) were applied. Fermentations were performed in triplicate with three different healthy donors (*n* = 3) and technical duplicates were also performed for each condition.

Initially, 300 mL sterile batch-fermentation vessels (200 ml working volume) were aseptically loaded with 180 mL of sterile GMM and sparged with O_2_-free N_2_ (15 mL/min) overnight to establish anaerobic conditions. Vessels were incubated at 37°C using a circulating water bath and culture pH was maintained at 5.4–5.6 using an automated pH controller (Fermac 260, Electrolab, Tewkesbury, UK), to reflect pH of the proximal colon. Then, each vessel was inoculated with 20 ml of fresh fecal slurry prepared at two different concentrations to determine the effects of low (0.1% v/v) and high inocula (1% v/v) on gut-microbiota growth. The effect of a range of iron/haem levels were explored during the fermentation: (1) GMM with 18 μM FeSO_4_ and 77 μM haem; or GMM without iron/haem supplementation (Figure 1A). Batch cultures were conducted for 48 h, and samples collected from each vessel at 0, 12, 24, 36, and 48 h for analysis of bacterial populations, organic acids and metal levels in the cell pellets and culture supernatants. Each growth condition was performed in biological triplicate (using 3 different donors) and technical duplicate (with the same donor).

### *In vitro* batch cultures (100 ml) for validation of mGMM

To compare the abilities of novel mGMM and standard GMM to support growth of gut microbiota in batch culture, 100 ml fermentation vessels (50 ml working volume) under the same pH and temperature conditions as described above, with a low final inoculum (0.1% v/v) level, were deployed. Fecal inocula from six different donors were used and each inoculum was applied to the two different culture media (GMM and mGMM) (Figure 1B). Batch cultures were operated for 36 h, and samples were collected from each vessel at 0 and 36 h for quantifying bacterial populations and for analysis of iron assimilation profiles during fermentation.

### *In vitro* batch cultures (250 ml) in mGMM with/without haem and/or inorganic iron

To determine the impact of iron supplementation on low-iron mGMM, 300 ml fermentation vessels (250 ml working volume of mGMM) were used using the same pH and temperature conditions as described above, with low-volume fecal inocula (0.1% v/v*)*. Deployment of the low-iron mGMM allowed a wider range (~36-fold) of total iron levels to be tested (from 5 to 180 μM). Iron supplements applied to the mGMM were as follow: (1) no additions; (2) 7.7 μM haem only; (3) 18 μM FeSO_4_ only; (4) 18 μM FeSO_4_ and 7.7 μM haem; (5) 77 μM haem only; (6) 18 μM FeSO_4_ and 77 μM haem; and (7) 180 μM FeSO_4_ only (Figure 1C). Fermentations were performed in triplicate using three different healthy donors and technical duplicates were conducted for each condition.

### Determination of metal content of media, cell pellets, and culture supernatants

Fermented aliquots (2 ml) were centrifuged at 10,000 *g* for 10 min. The resulting pellet and supernatant fractions were separated and then stored at −20°C. Metal analysis was performed on the pellet and supernatant fractions to assess assimilation of iron by the gut microbiota during fermentation. Briefly, the pellet and supernatant samples were digested in 735 μl of analytical grade nitric acid (65% pure) in tightly-lidded 15 ml Falcon tubes by overnight incubation at 80°C. After digestion, Milli-Q water was added to the samples to achieve a final volume of 10 ml and a final concentration of 5% nitric acid. Samples were filtered through 0.22 μm (pore size) PVDF-membrane syringe filters (Fisher Scientific Ltd., Loughborough, UK) before metal analysis. Finally, samples were analyzed for a suite of elements (Be, Ca, Co, Cu, Fe, Mn, Mo, Ni, Se, V, Zn) using the ICP multi-element standard solution XVI (Merk chemicals, Southampton, UK) by Inductively coupled plasma atomic emission spectroscopy (ICP-OES; Perkin Elmer Optima 3,000). Fresh media and components were assayed as above.

### Quantification of lactic acid and short-chain fatty acids

Collected samples (2 ml) were centrifuged at 10,000 *g* for 10 min and supernatants decanted into fresh 1.5 mL microtubes (Fisher Scientific Ltd., Loughborough, UK) and stored at −80°C for further analysis. Supernatant samples (400 μL) were then transferred to labeled 100×16-mm glass tubes (International Scientific Supplies Ltd., Bradford, England) containing 20 μL of 2-ethylbutyric acid (0.1 M, internal standard; Sigma-Aldrich, Poole, UK). Concentrated HCl (250 μL) and 1.2 ml diethyl ether (Sigma-Aldrich, Poole, UK) were added to each glass tube, and the samples vortexed for 1 min. Samples were then centrifuged at 2,000 *g* for 10 min. The diethyl ether (upper) layer of each sample was subsequently transferred to a labeled clean glass tube. The ether extract (200 μl) was then combined with 25 μl *N*-(*tert*-butyldimethylsilyl)-*N*-methyltrifluoroacetamide (MTBSTFA; Sigma-Aldrich, Poole, UK) in a gas chromatography (GC) screw-cap vial (Fisher Scientific Ltd., Loughborough, UK). Samples were left at room temperature for 72 h to allow lactic acid in the samples to completely derivatise.

An Agilent/HP 6890 gas chromatograph (Hewlett Packard, UK) equipped with a flame ionization detector (FID) was used for the quantification of organic acids. The column used was an HP-5MS (30 m × 0.25 mm) containing a 0.25-μm crosslinked (5%-phenyl)-methylpolysiloxane packing (Hewlett Packard, UK). Injector and detector temperatures were 275°C. Helium was the carrier gas (flow rate 6.5 ml/min; head pressure 90 MPa). A split ratio of 100:1 was used. Sample (1 μL) was injected into the column, which was programmed from 63°C for 3 min to 190°C at 10°C min^−1^ and held at 190°C for 3 min. Quantification of sample contents was enabled by calibration using a set of standards (lactic, acetic, propionic, butyric, valeric, isobutyric and isovaleric acids) with concentrations between 12.5 and 100 mM (Richardson et al., 1989).

### Quantification of total bacteria by flow cytometry-fluorescence *in situ* hybridization (*flow*-FISH)

Immediately after collection, culture samples (750 μL) were centrifuged at 10,000 *g* for 10 min at room temperature and the supernatant discarded. For bacterial fixation, pellets were re-suspended in 375 μL of PBS and 1,126 μL of cold 4% (*w*/*v*) paraformaldehyde (PFA) (Sigma-Aldrich, Poole, UK) and stored at 4°C for 4–6 h. After incubation, samples were washed twice with 1 ml of PBS to remove the PFA and resuspended in 300 μl PBS and 300 μL 99% ethanol. Samples were vortexed and stored at −20°C until further use. Prior to analysis by flow cytometry, pellets were washed with 500 μL of filtered ice-cold PBS and then washed with 150 μl of hybridization buffer (5 M NaCl, 1 M Tris–HCl at pH 8, formamide, Milli-Q water, and 10% SDS with the ratio of 180:20:300:499:1) and centrifuged at 10,000 *g* for 5 min. Pellets were then resuspended in 1 ml of hybridization buffer. Aliquots of 50 μl were mixed with 4 μl of 16S rRNA universal hybridization probes (50 ng·μL^−1^) (targeting total bacteria) and incubated at 35°C overnight. 16S rRNA universal hybridization probes used for the enumeration of total bacterial were a mixture of three different labeled oligonucleotides (1:1:1): Eub 338 I (GCTGCCTCCCGTAGGAGT), Eub 338 II (GCAGCCACCCGTAGGTGT) and Eub 338 III (GCTGCCACCCGTAGGTGT) (Daims et al., 1999). Oligonucleotide hybridization probes were labeled with the fluorescent Alexa Fluor 488 attached to the 5′ ends and were provided by Eurofins. Finally, samples were analyzed using a flow cytometer (Accuri C6, BD Biosciences, USA) and Accuri CFlow software in the Department of Food and Nutritional Sciences at University of Reading.

### DNA isolation from batch culture samples

Batch culture samples of 1 ml were centrifuged in Eppendorf tubes at 10,000 *g* for 10 min. Pellets were stored at −80°C. Cell pellets were defrosted on ice and washed with 0.5 ml of PBS. Then, DNA was extracted from the pelleted cells using the QIAamp DNA Stool kit (Qiagen, Hilden, Germany). Modifications in the lysis step of the supplied protocol were made to ensure lysis of Gram-positive bacteria. Briefly, each pellet was resuspended in 1 ml of InhibitEX buffer (provided in the kit) and suspensions were transferred to fresh 2 ml tubes containing 0.3 g of autoclaved zirconia beads (Fisher Scientific Ltd., Loughborough, UK). Bacterial cells were disrupted in a bead-beater at 6 m/s for 1 min (FastPrep-24 5G; MP BIO, Ca, USA). This step was repeated three times maintaining the samples on ice between cycles. Subsequent steps were performed according to the manufacturer’s protocol.

### 16S rRNA gene sequencing and data analysis

DNA samples were sent to the Animal and Plant Health Agency (Surrey, UK) for 16S rRNA gene sequencing. Aliquots of extracted DNA were amplified with universal primers for the V4 and V5 regions of the 16S rRNA gene. Primers U515F (5′-GTGYCAGCMGCCGCGGTA) and U927R (5′-CCCGYCAATTCMTTTRAGT) (Ellis et al., 2013) were designed to permit amplification of both bacterial and archaeal rRNA gene regions, while providing the best possible taxonomic resolution based on published information (Wang et al., 2007; Wang and Qian, 2009). Forward and reverse fusion primers consisted of the Illumina overhang forward (5′-TCGTCGGCAGCGTCAGATGTGTATAAGAGACAG) and reverse adapter (5′-GTCTCGTGGGCTCGGAGATGTGTAATAAGAGACAG), respectively. Amplification was performed with FastStart HiFi Polymerase (Roche Diagnostics Ltd., UK) using the following cycling conditions: 95°C for 3 min; 25 cycles of 95°C for 30 s, 55°C for 35 s, 72°C for 1 min; followed by 72°C for 8 min. Amplicons were purified using 0.8 volumes of Ampure XP magnetic beads (Beckman Coulter). Each sample was then tagged with a unique pair of indices and the sequencing primer, using Nextera XT v2 Index kits, and 2x KAPA HiFi HotStart ReadyMix using the following cycling conditions: 95°C for 3 min; 10 cycles of 95°C for 30 s, 55°C for 30 s, 72°C for 30 s; followed by 72°C for 5 min. Index-tagged amplicons were purified using 0.8 volumes of Ampure XP magnetic beads (Beckman Coulter). The concentration of each sample was measured using a fluorescence-based Quantifluor assay (Promega). Concentrations were normalized before pooling all samples, which were subsequently identified by their unique index combinations.

Sequencing was performed on an Illumina MiSeq with 2 × 300 base reads using V3 chemistry according to the manufacturer’s instructions (Illumina, Cambridge, UK). Data output were demultiplexed using in-built RTA software on the instrument.

Demultiplexed sequence data were then processed and analyzed using the open-source software pipeline Quantitative Insights Into Microbial Ecology 2 (QIIME 2) version 2019.4^1^ by applying the following workflow. First, forward and reverse reads were merged using the fastq-join function. Quality filtering was performed using 20 as the minimum Phred quality score. Sequences were then assigned to amplicon sequence variant (ASVs) through QIIME2’s built-in deblur command at 99% similarity threshold. Representative sequences for each ASV were assigned to different bacterial taxonomic levels using the bacterial Greengenes v13.8. For alpha and beta diversity tests, all samples were subsampled to an equal number of reads (7,062 reads per sample). The Microbiome Analyst web platform^2^ was used on processed sequence data to perform β-diversity and univariate analysis.

### Nucleotide sequence accession number

The sequence data obtained by sequencing of the V4-V5 region of the 16S rRNA gene have been submitted to the Sequence Read Archive (SRA) of NCBI^3^ under accession number PRJNA803451.

### Statistical analysis

Univariate analysis of variance and Tukey’s *post hoc* test were used to determine significant changes in total bacteria, SCFAs and alpha diversity among different iron regimes and time points using GraphPad Prism (version 8, GraphPad software, San Diego, California). Differences were considered to be significant when *p* < 0.05. The Microbiome Analyst web platform was used to perform permutational multivariate analysis of variance (PERMANOVA) for β-diversity analysis and differential abundance of taxa by univariate analysis using Mann–Whitney/Kruskal Wallis with an adjusted cut off value of 0.05 to identify significant taxa affected by iron treatment. Significant changes between iron treatments and control (no iron) at 36 h were determined.

## Results

### Low-inocula anaerobic batch cultures reveal iron-induced changes in the gut microbiota

In order to establish suitable conditions to explore the effects of iron regime in batch gut-culture systems, two final inocula densities were compared (1 and 0.1% v/v) using GMM with and without 18 μM inorganic iron together with 77 μM haem (Figure 1A). The hypothesis to be tested was that a lower inoculum density would result in a greater increase in total growth over time, providing better opportunities for alterations in the microbiota (in response to iron) to develop. In addition, use of a low fecal inoculum would minimize contributions of fecal contents (such as iron) to the culture conditions and thus help to limit cofounding factors.

**Figure 2:**
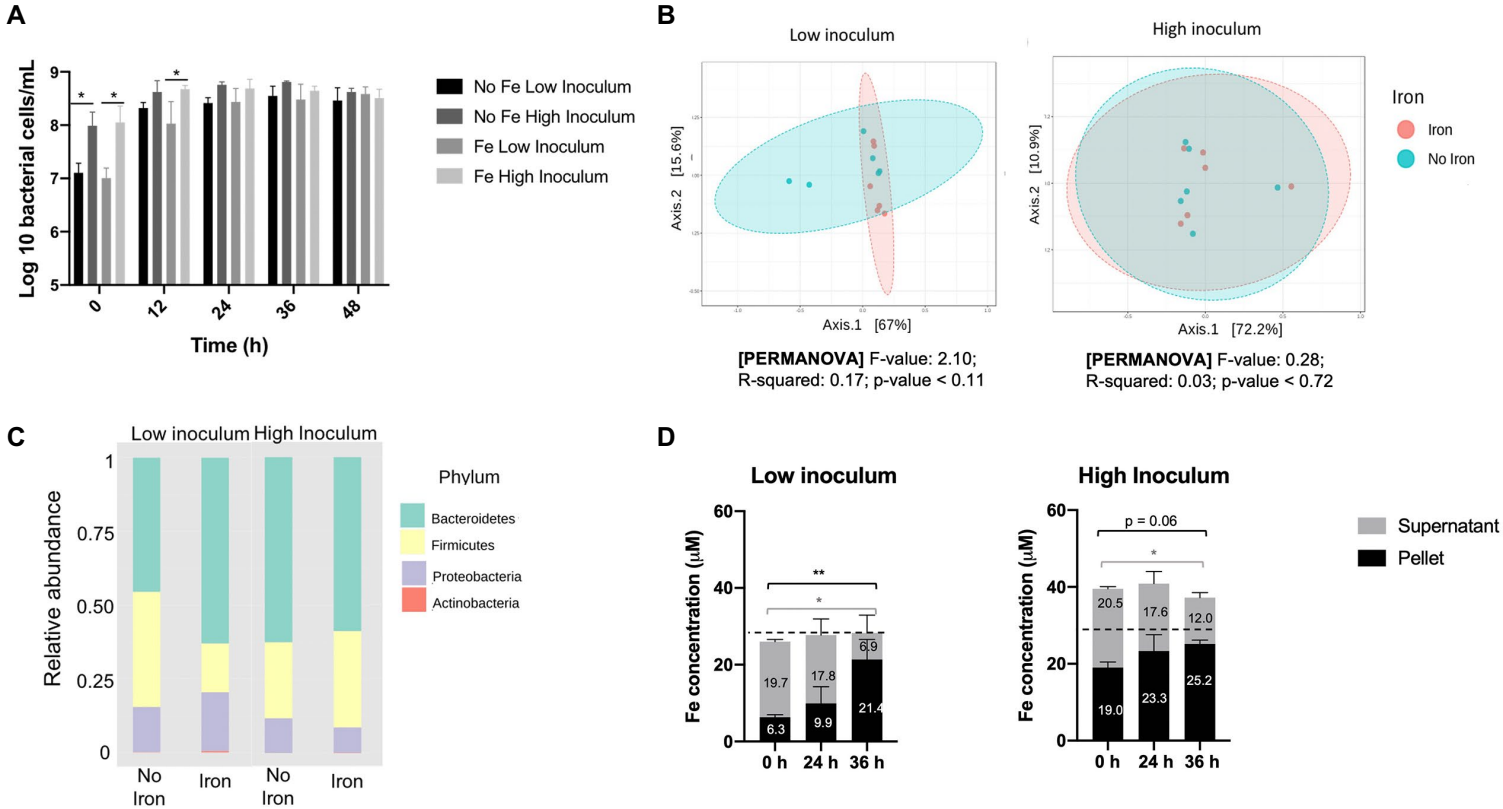
Impact of iron supplementation on the human gut microbiota at high and low inoculation levels using standard gut model medium (GMM). Single-stage anaerobic batch culture fermentations were performed under two iron regimes: GMM supplemented with 18 μM FeSO_4_ plus 77 μM haem; or GMM with no iron supplements. In addition, two different inocula levels were used: low (1% w/v); or high (10% w/v). Incubation was for 48 h and performed in triplicate with technical duplicates for each condition. Where shown, error bars indicate standard error. **(A)** Total bacterial counts over time, as determined by *Flow*-FISH. **(B)** Principal coordinates analysis of Weighted Unifrac distances of microbiota composition profiles (as determined by 16S rRNA gene amplicon sequencing) obtained at 36 h in the presence (pink) or absence (turquoise) of iron supplements. **(C)** Microbiota composition at phylum level in the absence or presence of iron supplements after 36 h. **(D)** Iron levels in cell pellet and culture supernatant fractions at 0–36 h. Data are only shown for the non-iron supplemented samples since iron-supplemented samples failed to show redistribution of iron from supernatant to pellet (possibly due to an iron saturation effect; data shown in Supplementary Figure S2). Significant differences between time points are denotated with asterisks: *p < 0.05, **p < 0.01. Dotted line indicates the 28 μM background iron in the GMM.

*Flow*-FISH analysis of total bacterial numbers at the 24 h time point showed that a high-fecal inoculum resulted in a modest 4.77− (±1.19) fold increase in bacterial counts whereas a far more substantial 27.5− (±6.59) fold change was achieved for the low-fecal inoculum (Figure 2A). As expected, the high and low inocula provided major differences in total bacterial numbers at baseline (10-fold, *p* < 0.05) and 12 h (2–5 fold, *p* < 0.05), although no significant differences in cell number were observed at the end (48 h) of growth (Figure 2A), indicating that similar final cell densities were achieved. Comparison of the effects of iron regime and inoculum density revealed no significant differences in SCFAs between low- and high-fecal inocula at the end of the growth (36–48 h stage) (Supplementary Figure S1). In addition, no significant correlation in microbiota composition with respect to iron regime was detected using principal coordinates analysis, either with a high or low inoculum (Figure 2B). However, the associated *p* value decreased from 0.7 at high inoculum to 0.1 at low inoculum, which reflects a trend toward clustering according to iron regime at the lower-inoculum level (pseudo-*F* = 2.10, *R* = 0.17, *p* < 0.11). Indeed, a higher abundance of Bacteroidetes (*p* = 0.09, FDR = 0.17) and lower ratio of Firmicutes (*p* = 0.06, FDR = 0.17) (ratio shift from 1.2:1 to 3.8:1, respectively) was observed when iron supplements were included in GMM under low-inoculum conditions (Figure 2C), but no such change was observed with the high inoculum.

ICP-OES analysis of the pellet and supernatant culture fractions obtained following growth in GMM without iron supplements showed accumulation of iron into the pellet fraction over the fermentation time-course under both low- and high-inocula conditions (Figure 2D). This is indicative of assimilation of iron from the medium by the microbiota as a result of growth. A total 12.6 μM iron (2.52 μmoles) was accumulated over 36 h by the cell pellet for the low inoculum while only 2.9 μM (0.58 μmoles) was accumulated with the high inoculum. These increases in pellet iron were closely matched by corresponding decreases in supernatant iron (Figure 2D). Furthermore, the amount of iron in the pellet at 0 h was three-times greater for the high inoculum than the low inoculum cultures (19.0 *cf.* 6.3 μM) resulting in a corresponding increase in total iron (Figure 2D). This difference (~12.7 μM iron) presumably reflects the levels of fecal inocula used, suggesting that 0.1 and 1% final inocula concentrations utilized each provided ~1.3 and 13 μM iron to the total iron contents presented in Figure 2D. Such fecal iron levels fall within the range previously reported for iron in feces (average concentration of 183 μg/g; ~3 mM) which would correspond to 3 and 30 μM in the 0.1 and 1% final inocula, respectively (Ingle et al., 2002). The increase in pellet iron observed in the low-iron, low inoculum conditions correlates to an average accumulation of 10.8×10^6^ iron atoms per bacterial cell – a value that is well within the range (~10^6^–10^8^) suggested by Rouf (1964) for five bacterial species, but higher than previously reported (~10^6^) by others (Jones et al., 1979; Abdul-Tehrani et al., 1999). In the iron-supplemented fermentation, any redistribution of iron from supernatant to pellet was less clear, possibly due to an iron saturation effect (Supplementary Figure S2). In summary, the above results indicate that the use of a low inoculum enhances the potential for detecting the impact of iron supplements on the gut microbiota in batch culture by increasing overall growth and minimizing introduction of fecal iron.

### Validation of a modified ‘Low-iron’ gut model medium

ICP-OES analysis showed that GMM, even without iron supplements, contains a relatively high level of iron at 28.3 (±0.26) μM. Generally, bacteria do not exhibit iron restriction until concentration falls below 5–10 μM (Andrews et al., 2003). Thus, it was considered necessary to modify the composition of GMM to generate a low-iron version that would allow comparison of low- and high-iron gut-culture conditions. Therefore, ICP-OES analysis was performed on each GMM component. In this way, mucin was identified as the main iron contributor (72%), followed by yeast extract (11%) and tryptone (5%) (Supplementary Figure S3). A ‘modified’ GMM (mGMM) was subsequently formulated within which the three high-iron components were replaced by low-iron alternatives (peptone and a mixture of trace nutrients) resulting in an iron content for mGMM of just 4.8 μM (as measured by ICP-OES). To determine whether mGMM supports microbiota growth similarly to standard GMM, batch gut culture fermentation results were compared for both media inoculated (at 1%) with fecal slurries from six different donors. 16S rRNA gene amplicon NGS showed no significant differences between microbiota in the two media (supplemented with 18 μM FeSO_4_ together with 77 μM haem) in terms of diversity and microbiota composition at 36 h (Supplementary Figure S4). This suggests that ‘modification’ of GMM does not significantly impact the growth of bacterial microbiota populations, at least when iron supplements are provided. This justifies the deployment of mGMM in the experiments below for investigating the impact of iron on the gut microbiota.

### Utilization of low-iron GMM (mGMM) to investigate the impact of iron and haem on the gut microbiota, *in vitro*

#### High levels of haem as sole iron supplement reduce growth

**Figure 3:**
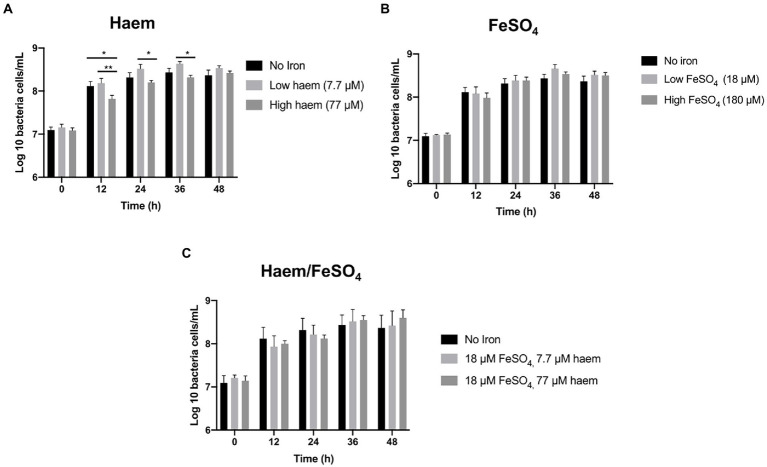
Effect of iron regime on gut microbiota *in vitro* growth in mGMM. Total bacterial counts in single-stage anaerobic batch culture fermenters were analyzed by flow cytometry-fluorescence *in situ* hybridization (Flow-FISH) to determine the effects of different sources and concentrations of iron in mGMM. **(A)** Effect of 7.7 and 77 μM haem on growth. **(B)** Effect of 18 and 180 μM FeSO_4_ on growth. **(C)** Effect of combining haem (7.7 and 77 μM) and 18 μM FeSO_4_. Error bars show SEM. Batch cultures were ran in triplicate inoculated with faecal samples from 3 different healthy donors (*n* = 3). Duplicates were performed for each condition in every case. Significant differences among treatments at the same time point are denotated with asterisks: *p < 0.05; **p < 0.01.

The mGMM was subsequently utilized, along with low-inoculation levels (0.1%), to examine the effect of seven distinct haem and inorganic iron regimes (Figure 1C) on the gut microbiota during growth in anaerobic batch cultures. Total bacterial counts for all regimes tested reached similar maximum final levels after 36 h incubation (Figure 3). Greatest numbers of total bacteria were achieved by supplementation with modest amounts of inorganic iron or haem (18 μM FeSO_4_ or 7.7 μM haem) after 36 h (8.66 log_10_ and 8.63 log_10_ cells per mL, respectively). In contrast, high haem concentration (77 μM) gave the lowest level of growth at 36 h (8.31 log_10_ cells per mL). The growth reduction caused by 77 μM haem was particularly marked (and significant *p* < 0.05) at 12 h with respect to the non-iron control and 7.7 μM haem conditions, at 2.2 and 2.5-fold, respectively (Figure 3A). Provision of 77 μM haem also caused a significant growth reduction at 24 and 36 h (2.2 and 1.8-fold, respectively) with respect to supplementation with just 7.7 μM haem (Figure 3A). These differences indicate that increasing haem levels from 7.7 to 77 μM results in significantly less growth of the gut microbiota. Conversely, bacterial growth was not markedly affected by high concentration of FeSO_4_ (180 μM) (Figure 3B) and, surprisingly, the effects of high concentration of haem (77 μM) were reversed when 18 μM FeSO_4_ was also provided (Figure 3C).

#### Haem in high concentrations induced a decrease in the production of acetate and propionate

**Figure 4:**
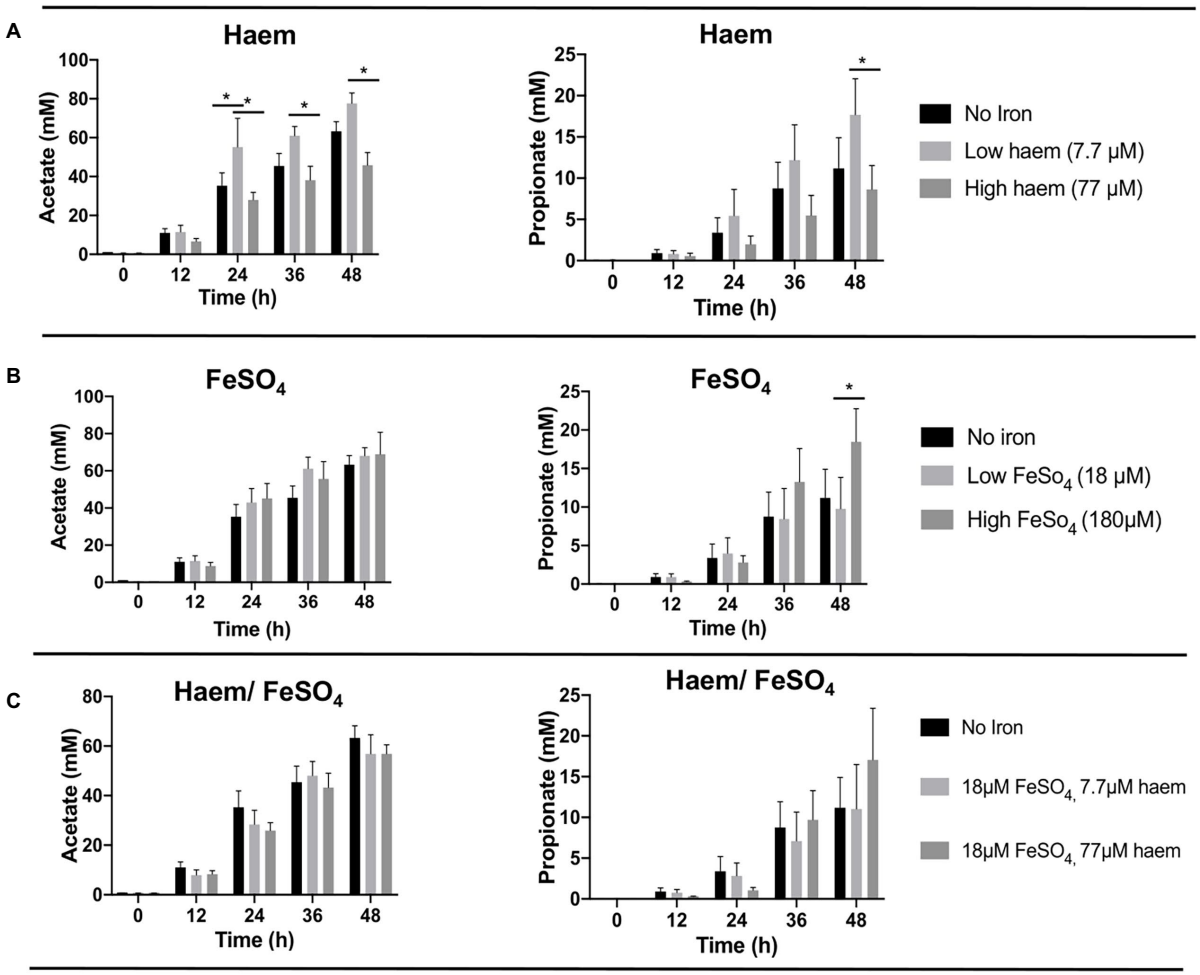
Acetate and propionate production from fermentation in single-stage anaerobic batch culture using different forms and concentrations of iron. **(A)** Haem, **(B)** FeSO_4_ and **(C)** combination of haem and FeSO_4_. Values are mean values of fermentations from 3 different healthy donors. Technical duplicates were performed for each condition in every fermenter. Significant differences (*p* < 0.05) among treatments at the same time point are denotated with an asterisk.

To determine whether the iron/haem regimes examined above had any impact on the production of key fermentation products of the gut microbiota, SCFAs levels accumulated in the culture medium were measured and compared (Figure 4; Supplementary Figure S5). As expected, SCFA concentrations were low in the fecal slurries (<1 mM or undetectable at baseline). In all cases, major increases in SCFAs were observed over the course of the fermentation (Supplementary Figure S5). Acetate was the main SCFA detected followed by propionate (Figure 4) and butyrate (Supplementary Figure S5). The highest concentration of acetate was obtained with low concentrations of haem (7.7 μM) after 48 h (77.7 mM ± 13.1) (Figure 4A). In contrast, 77 μM haem resulted in the lowest acetate concentration recorded at 48 h (45.8 mM ± 14.6), representing a significant (*p* < 0.05) decline in production with respect to that obtained with 7.7 μM haem (Figure 4A). For propionate, maximum production was detected with 180 μM FeSO_4_ at 48 h (18.5 mM ± 10.5) (Figure 4B) whereas a high concentration of haem (77 μM) caused the lowest levels recorded (8.63 mM ± 7.11) at the 48 h time point (Figure 4B). No significant differences were observed in butyrate production between treatments. Interestingly, provision of FeSO_4_, along with 77 μM haem, countered the effects of high haem such that no major SCFA differences were observed when both FeSO_4_ and haem were present (Figure 4C). Thus, the impact of iron/haem regime on SCFA production (Figure 4) resembles that seen for growth (Figure 3).

#### High concentrations of haem reduced gut microbiota diversity

**Figure 5:**
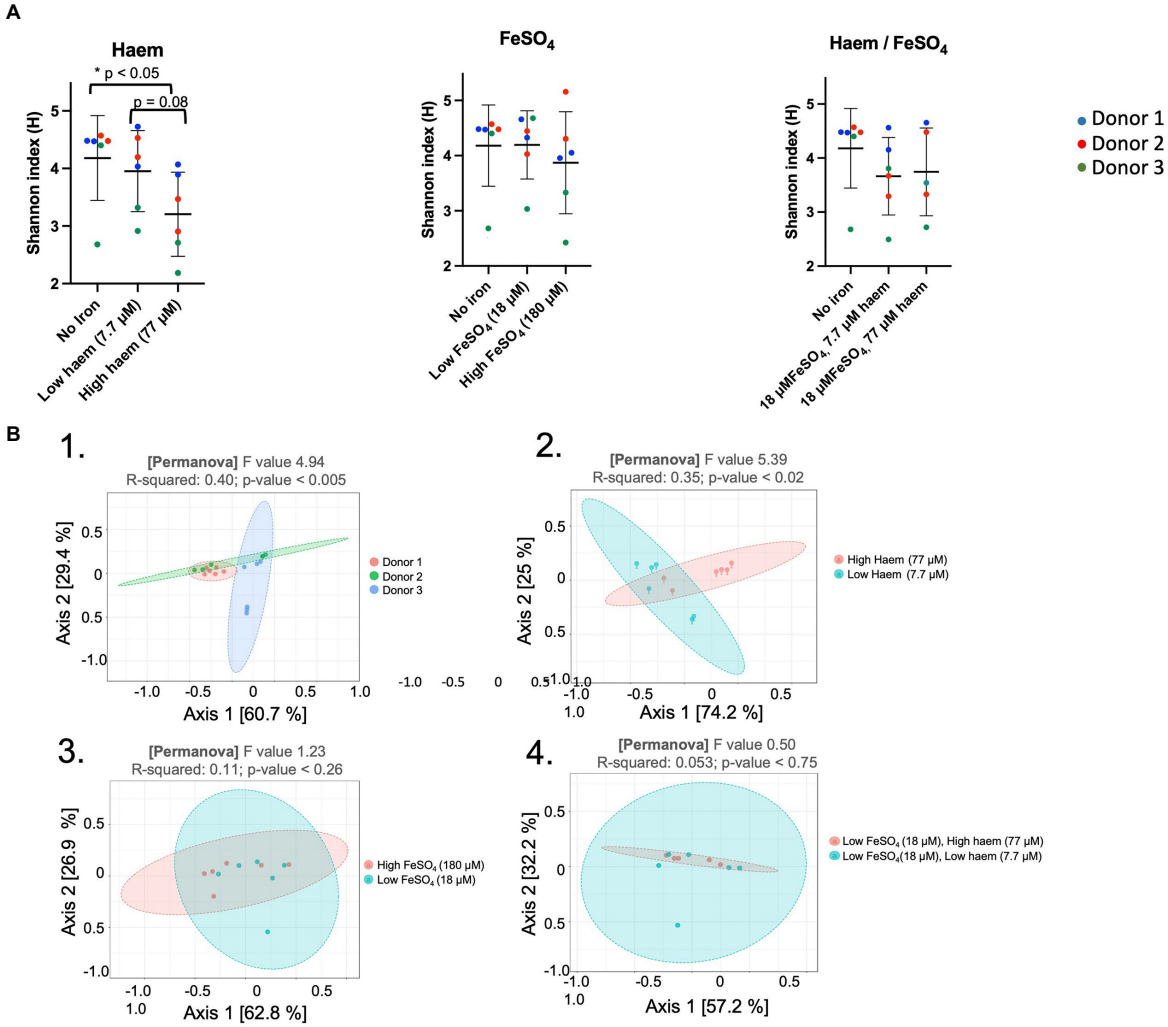
Impact of haem and inorganic iron on alpha and beta diversity. Microbiota composition was determined by 16S rRNA gene amplicon NGS. Samples were obtained following incubation in mGMM in the presence of 0, 7.7, or 77 μM haem, and/or 0, 18, or 180 μM FeSO_4_ after 36 h culture. Data from technical duplicate analyses of each of the 3 donors are presented. **(A)** Shannon diversity index for microbiota after 36 h of fermentation. Significant differences between the iron regimes are denotated with an asterisk (*p* < 0.05). **(B)** Principal coordinates analysis (PcoA) of Weighted Unifrac distances. PCoA was used to plot beta-diversity of the corresponding microbiota samples: from different donors (B.1); different haem concentrations (B.2); different FeSO_4_ concentrations (B3); and different combined concentrations of haem with FeSO_4_ (B.4).

The effect of the iron/haem regimes examined above (Figure 1C) on gut microbiota composition was determined by 16S rRNA gene amplicon NGS. This enabled alpha diversity of the microbiota to be determined using Shannon’s index whereby the number of species is measured according to their relative evenness (Figure 5A). Absence or low (18 μM) inorganic iron supplementation resulted in the highest overall diversity obtained (4.18 ± 0.74 and 4.20 ± 0.62, respectively). However, 77 μM haem as the sole iron-supplement significantly reduced alpha-diversity (3.20 ± 0.73) (*p* < 0.05) compared to mGMM without iron (4.18 ± 0.74), or mGMM with low haem concentration (3.95 ± 0.70) after 36 h of fermentation (Figure 5A). In contrast, there was little effect on alpha-diversity observed when FeSO_4_ was the sole iron supplement at 18 μM but there was a decrease at 180 μM, although this was not a significant effect (Figure 5A). Interestingly, the effect of 77 μM haem on diversity was partly counteracted when 18 μM FeSO_4_ was also provided (Figure 5C); this reflects counteractive effects of inorganic iron on the impact of 77 μM haem, as presented above, and indicates that the presence of FeSO_4_ mitigates loss of diversity caused by the presence of haem. Although neither 7.7 μM haem nor 18 μM FeSO_4_ alone had much impact on diversity, when these levels of haem and inorganic iron were combined there was a clear reduction in alpha diversity (0.5 units), although this was not significant.

Assessment of beta-diversity using principal coordinate analysis (PcoA) showed that microbiota samples could be grouped according to donor or to haem regime (Figures 5B-1, B-2). Indeed, Weighted Unifrac similarity matrices and subsequent permutational multivariate analysis of variance (PERMANOVA) revealed a significant correlation between microbiota composition and donor (pseudo-*F* = 4.94, *R* = 0.40, *p* < 0.005), and microbiota composition and haem concentration (pseudo-*F* = 5.40, *R* = 0.35, *p* < 0.02). However, no significant clustering was detected in response to FeSO_4_ level (*R* = 0.11, *p* < 0.26) or for haem/FeSO_4_ combination (*R* = 0.05, *p* < 0.75) (Figures 5B-3, 5B-4).

#### Proteobacteria growth is enhanced by high concentrations of haem in the medium

**Figure 6:**
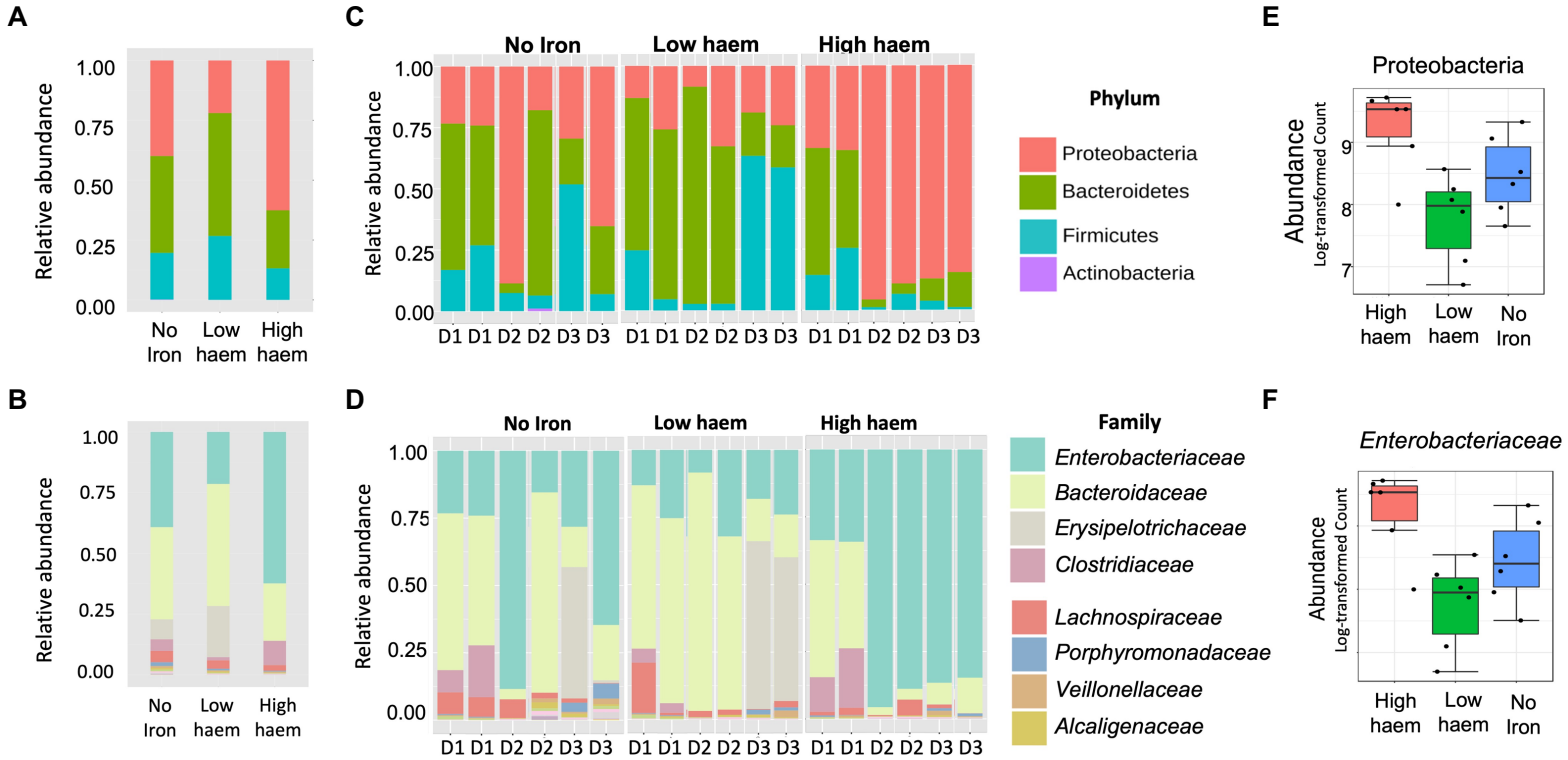
Impact of haem and inorganic iron on gut microbiota composition. Average **(A, B)** and individual **(C, D)** relative abundances of microbiota composition in response to haem addition (0, low 7.7 μM or high 77 μM) at phylum **(A, C)** and family levels **(B, D)** at 36 h. D1, D2, and D3 in figures **(C)** and **(D)** refer to the faecal donors. Relative abundance of taxa showing significant (*p* < 0.05) change in response to haem regime for the Proteobacteria phylum **(E)** and the family *Enterobacteriaceae* **(F)** are shown.

16S rRNA amplicon sequencing yielded a total of 1,319,500 reads, an average of 33,833 reads per sample (range 7,062 to 77,778 per sample) and a total of 1,204 OTUs. Total sequence reads used in this study were classified into 8 phyla. As expected, bacterial communities at time 0 h, in all cultures, were dominated by bacteria belonging to three phyla: Bacteroidetes (58.5 ± 6.71%), Firmicutes (36.1 ± 4.96%), and Proteobacteria (5.05 ± 1.99%) phyla (Supplementary Figure S6). Five other phyla (Actinobacteria, Cyanobacteria, Tenericutes, Verrumicrobia, and Synergistetes) were detected at lower (<1% each) levels. Univariate statistical comparisons at different taxonomic levels, performed to determine compositional shifts in response to the iron treatments, showed that the Proteobacteria phylum was significantly associated (*p* = 0.039) with haem (Figures 6A,C,E). Proteobacteria abundance was 3.4-fold overrepresented (71.3 ± 29.1%) in gut culture samples with high haem compared to those with lower haem concentrations (20.8 ± 9.44%). Conversely, abundance of the Bacteroidetes phylum declined 2-fold in the presence of high amounts of haem (28.3 ± 20.8%) compared to low haem (57.4 ± 20.1%), although this difference was not significant after FDR correction (*p* = 0.1) (Figure 6A). Haem-induced expansion of the Proteobacteria was almost entirely due to raised levels of unclassified *Enterobacteriaceae* family members (Figures 6B,D,F). Although data were analyzed at deeper taxonomic levels, no significant differences were observed at OTU or genus level after FDR correction.

Provision of FeSO_4_, either with or without haem, did not cause any significant difference in microbiota composition (Supplementary Figure S7). Thus, effects induced by high concentrations of haem were not detected when FeSO_4_ was also included in the medium. Provision of 180 μM FeSO_4_ (without haem) caused a 1.8-fold increase in *Bacteroidaceae* compared to low FeSO_4_, although this did not reach statistical significance (*p* = 0.13, FDR = 0.58) (Supplementary Figure S7).

## Discussion

### Advantages of the batch culture approach

The research reported here explores the impact of haem- and non-haem-iron dietary regime on the human gut microflora using anaerobic batch culture fermenters. Previous *in vitro* studies have also considered the impact of iron on the gut microbiota, but these have largely utilized continuous culture models (Dostal et al., 2013, 2015; Kortman et al., 2016). However, the continuous culture approach is time consuming (typically over one month per culture) and is thus restricted to testing a limited number of interventions and doses (Marsh, 1995; Macfarlane et al., 1998). In contrast, batch cultures allow rapid, simultaneous experiments to be performed that enable investigation of a wider range of conditions in parallel (McDonald, 2017). Thus, the use of batch cultures is recognized as the most appropriate approach for an initial assessment of multiple dietary conditions, which subsequently allows selection of regimes of interest for further exploration in more complex experimental systems. For this reason, batch cultures have been successfully and extensively employed for studying the impact of fermentable substrates on the gut microbiota (Sarbini et al., 2013; Frost et al., 2014; Mateos-Aparicio et al., 2016). However, such systems have been little exploited, thus far, to study the impact of micronutrients on the gut microbiota and have not been previously used to compare the impact of different doses of haem and/or non-haem iron on the gut microbiota.

### Adapting the batch culture approach for exploring iron regime – low inoculum

Traditionally, a standard quantity (10% w/v) of fecal material is used to inoculate anaerobic batch gut cultures (1% v/v final inocula concentration) (Khalil et al., 2014; Likotrafiti et al., 2014; Wang et al., 2019). However, this relatively high inoculation level allows only a modest (up to 4-fold) overall expansion of the gut microbiota (Figure 2A) which limits the potential for any impact of nutrient regime to be realized over the short-term fermentation period employed. In the research presented here, the impact of a 10-fold lower (0.1% v/v) final fecal inoculum was investigated and shown, as anticipated, to result in a much higher magnitude of growth over the fermentation time-course (up to 27.5 fold), providing greater opportunity for alterations in the microbiota in response to iron to be observed (Figure 2). In addition, the lower inoculum level greatly reduced adventitious introduction of iron from fecal matter (the inoculum), thus providing a greater control of iron regime (Figure 2D). Consequently, the low-inoculum condition was found to enable an iron-regime-induced change in gut microbiota composition: a 2.3-fold increase in Firmicutes, and 1.3- and 1.4-fold reductions in Proteobacteria and Bacteroidetes, respectively, in response to lack of iron and haem supplementation (reduction from 123 to 28 μM total Fe). Such changes were not apparent when the batch cultures were treated with a high dose inoculum (Figure 2C). Although these iron-depletion-induced changes in the microbiota were not significant, previous studies have observed significant reductions in Bacteroidetes levels in response to reduced iron supply. Dostal et al. (2013), using a continuous culture model, found a 2.8-fold reduction in the *Bacteroidaceae* family (members of the Bacteroidetes) in response to a reduction in iron levels (from 27.9 to 16.1 μM). In addition, *in vivo* studies on rats and infants have reported that lowering iron in the diet reduces Bacteroidetes levels by 4.5-fold (Krebs et al., 2013; Dostal et al. (2012) Ijssennagger et al., 2012b).

### Improving the batch culture approach for exploring iron regime – low iron

The failure to observe any iron-induced changes in the gut microbiota that achieved significance could be due to relatively high background iron levels (≈28 μM) in the GMM culture medium since such high pre-existing levels may well limit any impact of additional iron supply on the gut microbiota. To overcome this issue and impose an iron-depleted growth condition, a modified GMM (mGMM) was designed with a low total-iron content (<5 μM) but with sufficient levels of other essential micronutrients. It should be noted that others have also attempted to reduce iron available in gut culture studies. Such approaches have included pre-treating the medium with Chelex® 100, which reduced iron levels by ~1.7-fold to 16 μM (Dostal et al., 2013); however, levels achieved were more than threefold higher than those generated in the current study. Another strategy employed the ferrous-iron chelator 2,2′-dipyridyl at (150–300 uM) (Dostal et al., 2013; Parmanand et al., 2019). However, this chelator fails to remove iron from the medium (it simply restricts its availability), is lipid soluble and thus passes through biological membranes, and has affinity for metals other than iron (Wu et al., 2012; Constable and Housecroft, 2019). For these reasons, it can be difficult to associate any effects observed to a specific impact on iron availability. The ferrous-iron chelator, bathophenanthroline disulphonic acid (at 70 μM), has also been deployed to simulate low iron conditions in gut culture (Parmanand et al., 2019), and similar issues apply.

The low-iron mGMM utilized in the studies described here was developed by substituting three components of GMM that are major contributors of its high 28 μM background iron level. Thus, tryptone and yeast extract were replaced with peptone and micronutrient solutions, and mucin was excluded. Mucin is the principal glycoprotein in the intestine and is consumed by the microbiota (Marcobal et al., 2013). However, GMM is designed to carry an excess of carbohydrate to ensure that it is characteristic of the conditions of the proximal colon where saccharolytic metabolism predominates (Macfarlane et al., 1998). Thus, when GMM and mGMM batch culture fermentations were compared (under iron sufficiency), no significant differences were observed in terms of microbiota composition and diversity (Supplementary Figure S4).

### Negative effect of high haem on the human gut microbiota

The above adjustments in the batch culture system facilitated subsequent exploration of the impact of haem and non-haem iron (ferrous sulfate) on the gut microbiota. Thus, seven distinct haem/non-haem-iron regimes were compared, in parallel (Figure 1C). Supplementation with haem at high concentration (77 μM) elicited the greatest observed impact on the gut microbiota, causing a reduction in total bacterial growth (up to 2.2-fold), a lower alpha-diversity (23% reduction in Shannon Index) and a significant increase in *Enterobacteriaceae* (3.4-fold). Similar results were found in animal studies (Ijssennagger et al., 2012; Constante et al., 2017). In one case, providing rats with a high haem diet resulted in decreased gut microbiota α-diversity and an increase in Proteobacteria which was specifically associated with raised *Enterobacteriaceae* levels (at the apparent expense of Firmicutes) (Constante et al., 2017). In another case, a high haem diet in mice caused a two fold increase in Proteobacterial levels (including a 1.8-fold increase in γ-Proteobacteria), as well as a marked shift away from Firmicutes toward Bacteroidetes (Ijssennagger et al., 2012).

Haem is considered to be a favorable source of dietary iron due to its relatively high absorption rate (20%–30%), which is approximately double that of non-haem iron (Anderson et al., 2005). Despite this high rate of haem uptake from the diet, most remains unabsorbed and thus persists into the intestine where it may impact upon the gut microbiota. Pathogenic enterobacteria, which require iron for host infection, possess specific acquisition systems for utilization of haem as an iron source (Contreras et al., 2014). This may explain why a provision of haem at high concentration favored growth of *Enterobacteriaceae* (Figure 6F), which includes numerous enteropathogens and pathobionts (Donnenberg, 2019). The haem-induced increase in *Enterobacteriaceae* would be expected to reduce niche availability for other dominant groups of bacteria and may thus explain the reduction in Bacteroidetes and Firmicutes (Figure 6A), and the resulting reduction in diversity (Figure 5A). Exogenous haem can also exert toxic effects on bacteria and the host caused by processes such as membrane disruption, DNA damage due to the hydrophobic structure of haem and cytotoxic effects in the gut (Anzaldi and Skaar, 2010). Furthermore, it appears that Gram-positive bacteria are particularly sensitive to haem toxicity (Nitzan et al., 1994). Such effects could be, at least partly, responsible for the observed reduction in total bacteria under high-haem conditions (Figure 3A) and the concomitant reduction in SCFA production (Figure 4A).

Conversely, the family *Bacteroidaceae* was overrepresented in low-haem conditions (52.3% ± 29.9) compared to high haem (19.8% ± 20.4) or no iron (36.7% ± 27.7). *Bacteroides* spp. are considered as haem auxotrophs that cannot synthesize the tetrapyrrole macrocycle protoporphyrin IX (PPIX) needed to produce haem (Rocha and Smith, 2010) and therefore require either exogeneous haem, or PPIX and iron, to survive. However, in the absence of haem supplementation, *Bacteroides* spp. were abundant after 36 h growth (36.7% ± 27.7). This indicates that, under the culture conditions employed, members of this genus did not suffer from any general growth restriction caused by lack of haem (or PPIX and iron). Possible explanations for this observation include: trace-levels of haem within the fecal inoculum; endogenous PPIX production; and scavenging of haem/PPIX from other elements of the microbiota, as previously reported (Halpern and Gruss, 2015; Zhu et al., 2020). In summary, our *in vitro* studies support previous work indicating that a high haem diet negatively impacts gut health.

### Impact of non-haem iron on the human gut microbiota

Interestingly, negative effects imposed by high haem were counteracted when 18 μM FeSO_4_ was also applied to the medium (Figures 4, 5). This may be because the addition of ferrous iron can provide a supplementary iron source supporting species lacking haem-acquisition capacity, allowing them to better compete against haem-utilizing members of the gut microbiota. Given the high background of adventitious iron (~28 μM) in GMM, it is unlikely that addition of high haem to this medium would have generated the effects observed in mGMM. This highlights the advantages of mGMM for studying the impact of iron on the microbiota.

Surprisingly, supplementation with FeSO_4_ as sole iron supplement in mGMM failed to generate any significant differences in the gut microbiota when the low (5 μM) and higher (23 and 185 μM) non-haem iron conditions were compared. However, although statistical significance was not achieved (Wilcoxon rank test *p* > 0.05), fermentations with high total levels of non-haem iron (185 μM) showed a higher abundance of Bacteroidetes (1.8 and 2.9-fold) and lower abundance of Proteobacteria (1.3 and 1.28-fold) compared to the 23 μM and 5 μM non-haem iron condition, respectively (Supplementary Figure S7). Bacteroidetes members are propionate producers in the gut and positive correlations between propionate and some Bacteroidetes genera such as *Bacteroides* and *Prevotella* have been detected in other studies (Salonen et al., 2014; Aguirre et al., 2016). Thus, an increase in *Bacteroides* spp. during the fermentations would explain the significant increment (~ 1.8-fold) in propionate production after 48 h of fermentation with 180 μM FeSO_4_ compared to non-iron and low FeSO_4_ supplementation (Figure 4B).

## Conclusion

In summary, this study highlights an improved approach in the application of anaerobic batch culture fermenters systems in the *in vitro* investigation of the impact or iron on the gut microbiota: the use of low fecal inocula and a low-iron gut model medium. The results presented here suggest that high levels of dietary haem are harmful to gut health, more so than FeSO_4._ The presence of haem at high concentration induced an increase in levels of potentially harmful enterobacteria at the expense of ostensibly beneficial bacterial groups, which is indicative of a detrimental health impact (Mahnic et al., 2020; Baldelli et al., 2021). In addition, high haem levels induced a reduction in gut-microbiota diversity which is a further adverse gut health indicator (Manichanh et al., 2006; Giloteaux et al., 2016; Dahl et al., 2017). Thus, results reported here indicate that a high haem diet may have a deleterious impact on gut health.

## Data availability statement

The datasets presented in this study can be found in online repositories. The names of the repository/repositories and accession number(s) can be found below: https://www.ncbi.nlm.nih.gov/, PRJNA803451.

## Author contributions

SA, GW, GG, DP, and AW: conceptualization. SA, GG, GW, AM-M, and AS: methodology. AM-M: data curation, software, and writing-original draft preparation. AM-M, AS, and AD: formal analysis. AM, AS, and AD: investigation. SA, GG, GW, and AS: writing-review and editing. All authors have read and agreed to the published version of the manuscript. All authors contributed to the article and approved the submitted version.

## Funding

This work was supported by the Diet and Health Research Industry Club (DRINC) of the UK Biotechnology and Biological Sciences Research Council (BBSRC) (BB/N021800/1).

## Conflict of interest

DP was employed by Vifor Pharma UK Limited.

The remaining authors declare that the research was conducted in the absence of any commercial or financial relationships that could be construed as a potential conflict of interest.

## Publisher’s note

All claims expressed in this article are solely those of the authors and do not necessarily represent those of their affiliated organizations, or those of the publisher, the editors and the reviewers. Any product that may be evaluated in this article, or claim that may be made by its manufacturer, is not guaranteed or endorsed by the publisher.

## Footnotes

^1^ http://qiime2.org

^2^ https://www.microbiomeanalyst.ca/

^3^ https://www.ncbi.nlm.nih.gov/sra
