# Supplementary material for: Impact of inorganic iron and haem on the human gut microbiota; An in vitro batch-culture approach
Source: Front Microbiol. 2023 Feb 23;14:1074637. doi: 10.3389/fmicb.2023.1074637 (PMC9995831; doi:10.3389/fmicb.2023.1074637)
Supplement: Supplementary file 1 [file Data_Sheet_1.PDF]

Supplementary Material

Supplementary Table S1: Composition of Gut Model Medium (GMM) and modified Gut Model Medium (mGMM) used in anaerobic batch cultures studies on the effect of iron on gut microbial fermentation. Mineral and vitamin solution content used to supplement the mGMM

	**GMM g/L**	**mGMM g/L**	**Mineral solution (100x) g/L**	
Tryptone	5		Na_3Citrate.$2H_2O$ (pH 6.5)	2.1
Peptone water	5	10	$MnSO_4.2H_2O$	0.093
Yeast extract	4.5		CoCl.$6H_2O$	0.1
Casein	3	3	$ZnSO_4.7H_2O$	0.17
Mucin	4		$CuSO_4.5H_2O$	0.087
Pectin	2	2	$AlK(SO_4)_2$.12H2O	0.02
Xylan	2	2	H_3BO_3	0.01
Arabinogalactan	2	2	Na_2MoO_4	0.1
Starch	5	5	$NiSO_4$	0.053
Guar gum	1	1	Na_2SeO_3	0.2
Inulin	1	1	V (III) Cl	0.01
KH_2PO_4	0.5	0.5	$Na_2WO_4.2H_2O$	0.01
$K_2HPO_4.3H_2O$	0.5	0.5	**Vitamin solution (100x) g/L**	
$NaHCO_3$	1.5	1.5	biotin	2
KCl	4.5	4.5	folic acid	2
NaCl	4.5	4.5	pyridoxine HCl	10
$MgSO_4.7H_2O$	1.25	1.25	thiamine HCl	5
$CaCl_2.2H_2O$	0.15	0.15	riboflavin 5 mg	5
Cysteine-HCl	0.8	0.8	nicotinic acid	5
ox-bile	0.4	0.4	DL-calcium pantothenate	10
Vitamin K	10	10	vitamin B12	0.5
Tween 80	1	1	p-aminobenzoic acid	5
			lipoic acid 5 mg	0.01
			Menadione	1

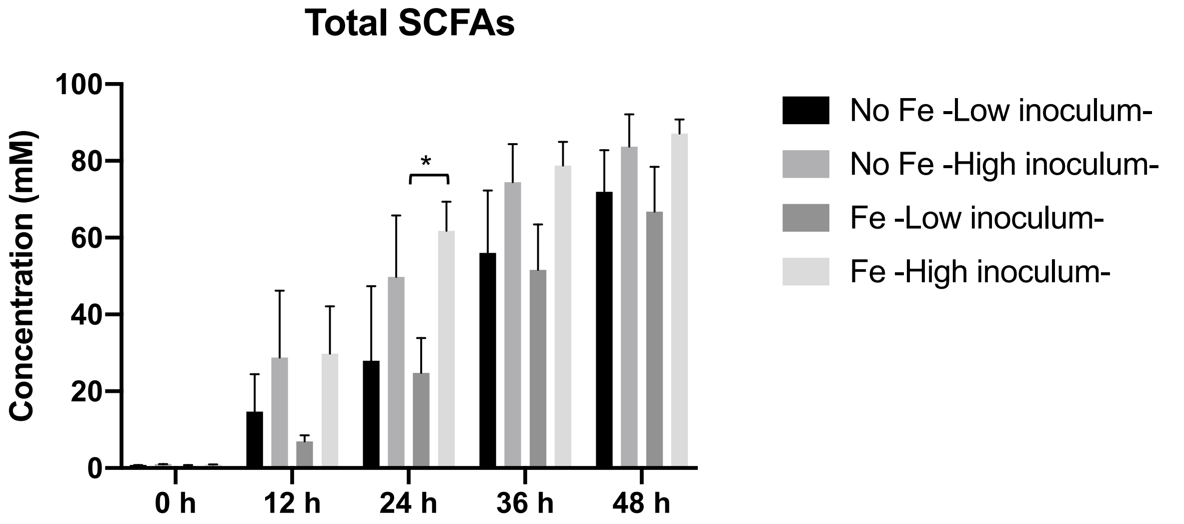

Supplementary Figure S1: Short Chain Fatty Acid production from fermentation in single-stage batch culture inoculated at two different densities and in presence or absence of iron supplementation. Samples were obtained at different time points (0h, 12 h, 24 h, 36 h and 48 h) from cultures grown in GMM supplemented, or not, with iron (18 µM $FeSO_4$ and 77 µM haem) and inoculated at 2 different densities (low 1% and high 10%). Values are mean values of fermentation from 3 different healthy donors. Technical duplicates were performed for each condition. Significant differences ($p < 0.05$) among time points are denotated with an asterisk.

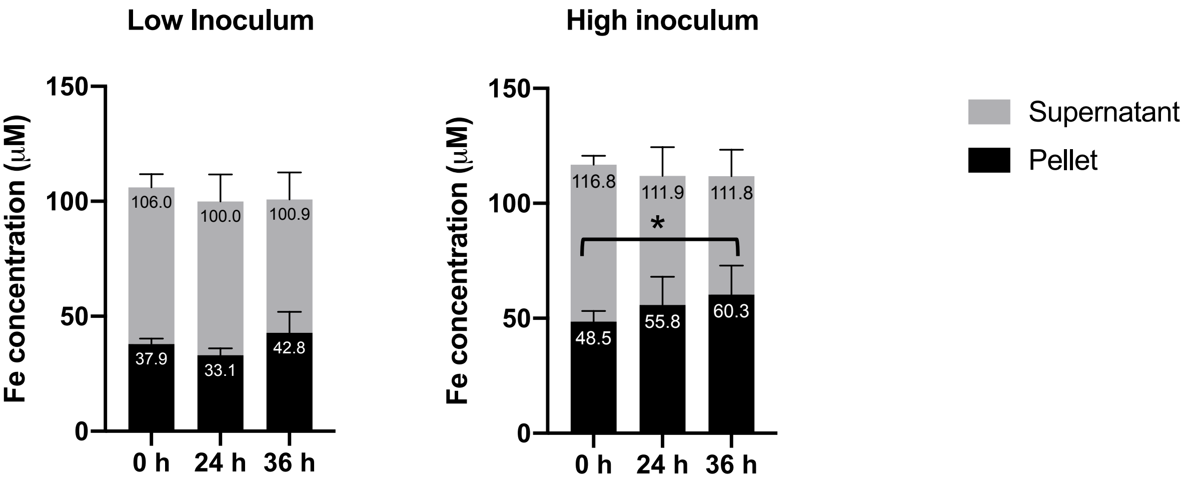

Supplementary Figure S2: Iron distribution in cell pellet and culture supernatant fractions at 0-36 h at high and low inoculation levels. Data from single-stage anaerobic batch culture fermentations supplemented with iron (18 µM $FeSO_4$ and 77 µM haem) and inoculated at 2 different ratios (low 1% and high 10%). Values are mean values of fermentation from 3 different healthy donors. Technical duplicates were performed for each condition. Significant differences ($p < 0.05$) among time points are denotated with an asterisk.

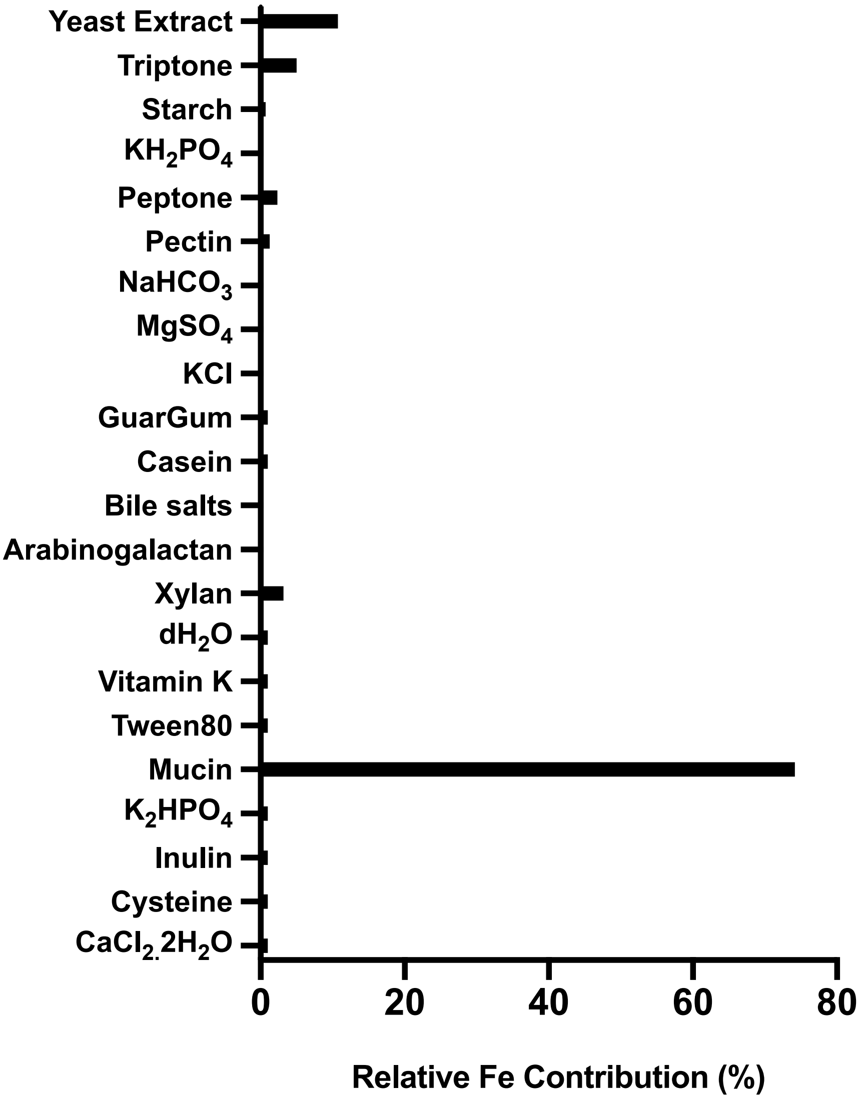

Supplementary Figure S3: Gut model growth medium components and relative iron contributions. Mucin (72%), yeast extract (11%) and tryptone (5%) were identified as the main iron contributors to the gut model medium.

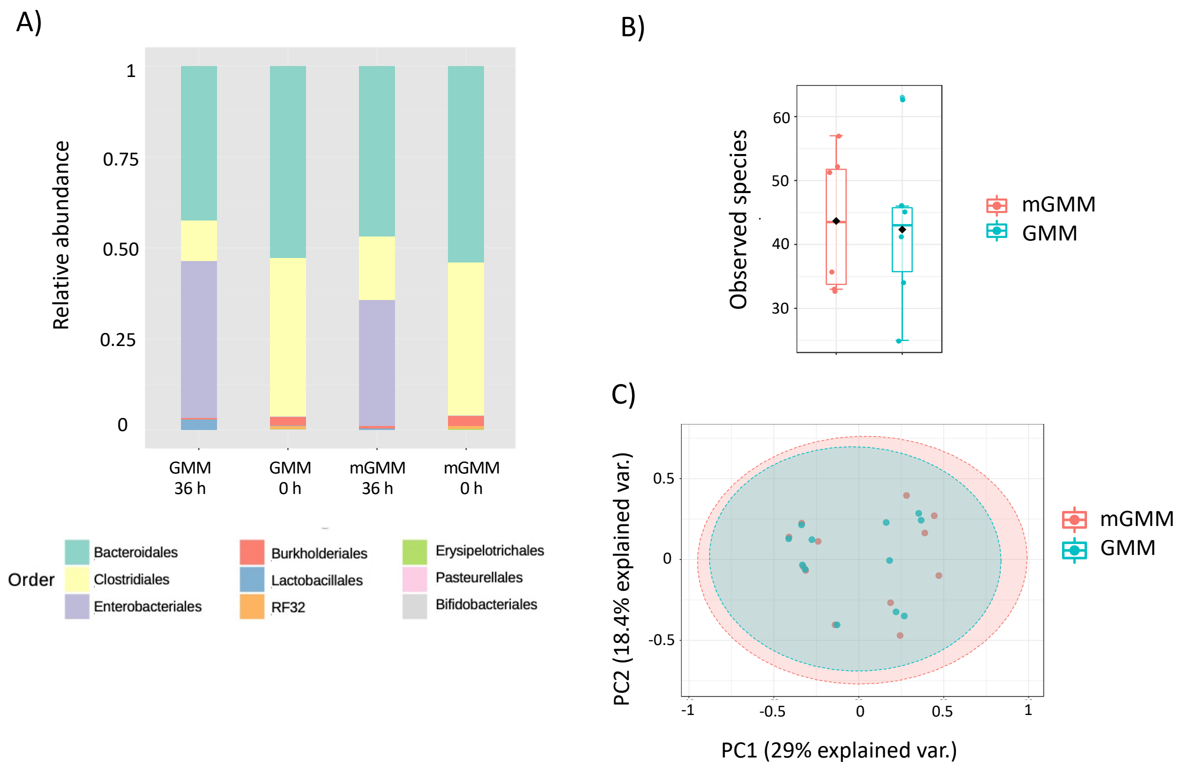

Supplementary Figure S4: Impact of GMM and mGMM on microbiota composition and diversity. A) Microbiota composition by 16S rRNA sequencing of fermented samples at baseline and after 36 h of fermentation in standard gut model medium (GMM) or modified GMM (mGMM). Microbial relative abundances (%) at order level. B) Observed species after 36 h of fermentation in GMM or mGMM. C) Principal coordinates analysis of Weighted Unifrac distances. PCoA was used to plot beta-diversity of fermented samples from different mediums (GMM or mGMM).

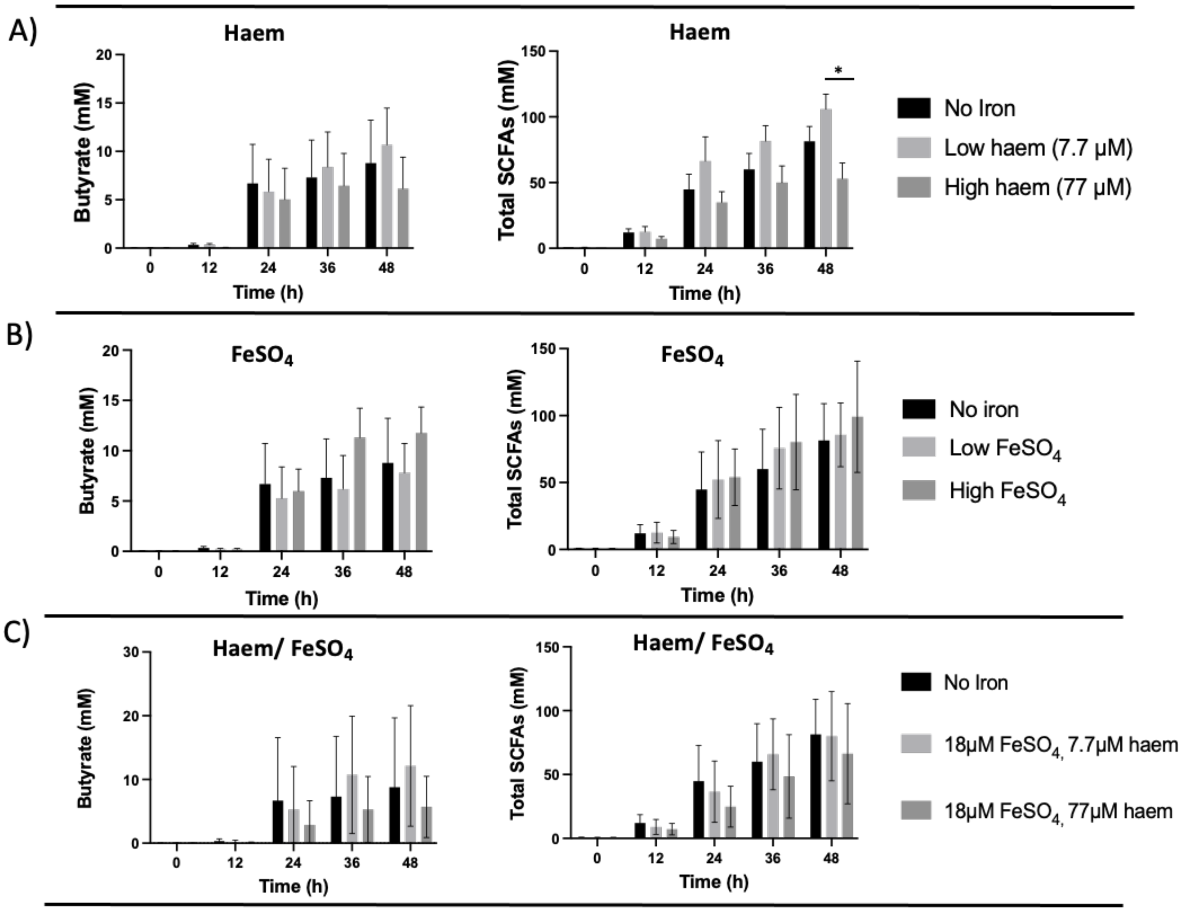

Supplementary Figure S5: Butyrate and total SCFAs production from fermentation in single-stage batch culture using different forms and concentrations of iron: A) haem, B) $FeSO_4$ and C) combination of haem and $FeSO_4$. Values are mean values of fermentation from 3 different healthy donors (n=3). Duplicates were performed for each condition in every batch. Significant differences among treatments at the same time point are denotated with an asterisk * ($p < 0.05$).

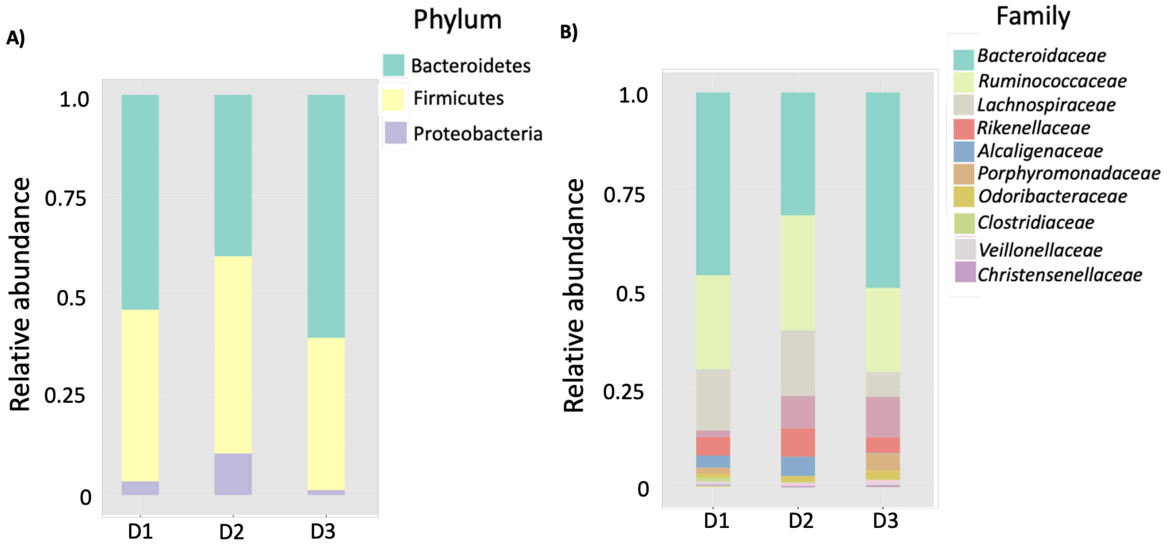

Supplementary Figure S6: Composition of the gut microbiota of faecal donors at baseline. Relative abundances of microbiota composition of faecal donors at baseline (time 0 h) at: A) Phylum level; and B) Family level. D1, D2 and D3 indicate distinct faecal donors.

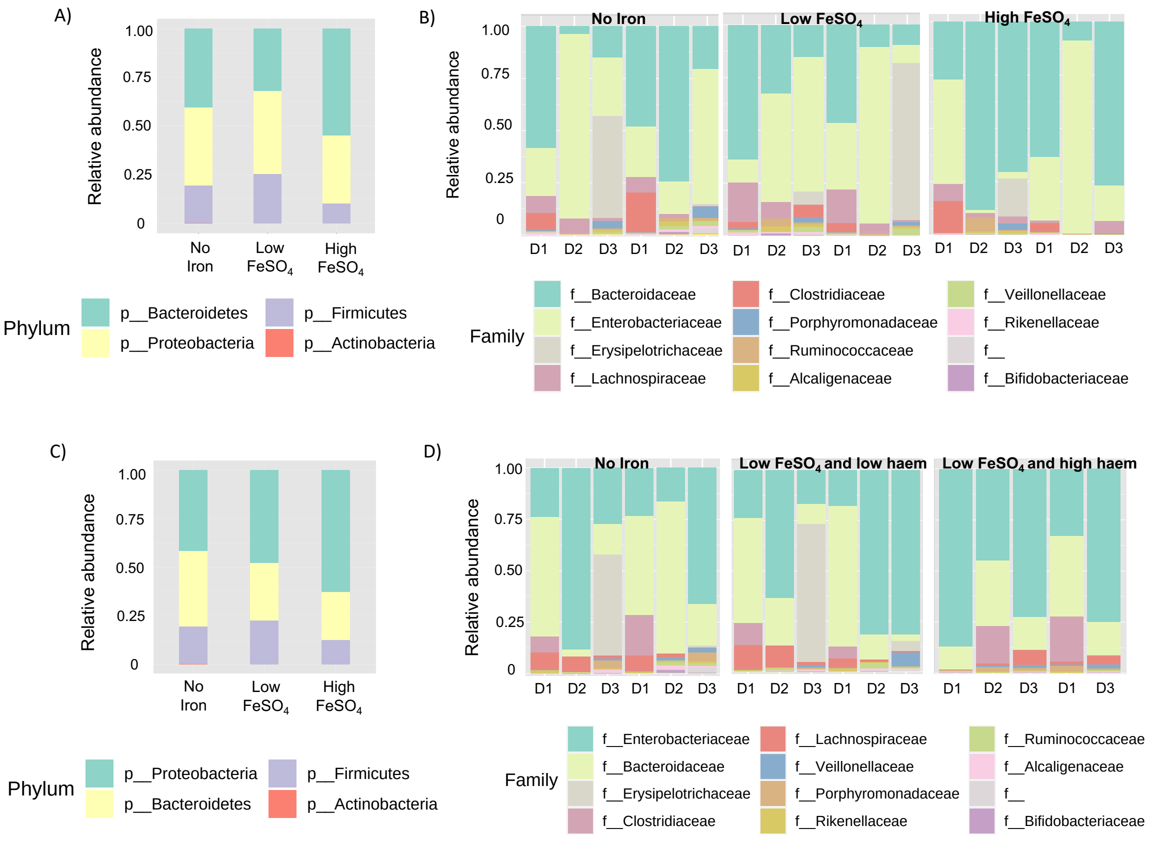

Supplementary Figure S7: Impact of $FeSO_4$, and combination of $FeSO_4$ and haem on gut microbiota composition. (**A,B**) Relative abundances of microbiota composition of samples fermented under different $FeSO_4$ concentrations (0 µM, no iron; 18 µM, low $FeSO_4$; and 180 µM, high $FeSO_4$) at phylum (**A**) and family level (**B**). D1, D2 and D3 refer to faecal donors. **(C,D)** Relative abundances of microbiota composition of samples fermented under different $FeSO_4$ and haem concentrations (0 µM $FeSO_4$ and 0 µM haem, no iron; 18 µM $FeSO_4$ and 7.7 µM haem, low $FeSO_4$ and low haem; 18 µM $FeSO_4$ and 77 µM haem, low $FeSO_4$ and high haem. (**D**). D1, D2 and D3 refer to faecal donors.
